# Hyunsoonleella sp. HU1-3 Increased the Biomass of *Ulva fasciata*

**DOI:** 10.3389/fmicb.2021.788709

**Published:** 2022-01-31

**Authors:** Han Wang, Ali Mohamed Elyamine, Yuchun Liu, Wei Liu, Qixuan Chen, Yan Xu, Tao Peng, Zhong Hu

**Affiliations:** ^1^Key Laboratory of Resources and Environmental Microbiology, Department of Biology, Shantou University, Shantou, China; ^2^Heyuan Polytechnic, Heyuan, China; ^3^Southern Marine Science and Engineering Guangdong Laboratory, Guangzhou, China

**Keywords:** *Ulva fasciata*, growth stage, *Hyunsoonleella* sp. HU1-3, bacterial treatment, promoting-plant-growth-bacteria

## Abstract

Green algae are photosynthetic organisms and play an important role in coastal environment. The microbial community on the surface of green algae has an effect on the health and nutrition of the host. However, few species of epiphytic microbiota have been reported to play a role in promoting the growth of algae. In this study, 16S rDNA sequencing was used to study the changes of microbial composition on the surface of *Ulva fasciata* at different growth stages. Some growth promoting bacteria were identified. The possible growth-promoting behavior of the strains were verified by co-culture of pure bacteria obtained from the surface of *U. fasciata* with its sterile host. Among the identified species, a new bacterial species, *Hyunsoonleella* sp. HU1-3 (belonging to the family Flavobacteriaceae) significantly promoted the growth of *U. fasciata*. The results also showed that there were many genes involved in the synthesis of growth hormone and cytokinin in the genome of *Hyunsoonleella* sp. HU1-3. This study identified the bacterium *Hyunsoonleella* sp. HU1-3 for the first time, in which this bacterium has strong growth-promoting effects on *U. fasciata*. Our findings not only provide insights on the establishment of the surface microbiota of *U. fasciata*, but also indicate that *Hyunsoonleella* sp. HU1-3 is one of the important species to promote the growth of *U. fasciata*.

## Introduction

Epiphytic microbiota refers to a specific community harboring the surface of plants and does not parasitize or interfere with the growth and development of its host (Gopal and Gupta, 2016). The microbiota can be found on terrestrial plants, marine macroalgae (Short et al., 2015) and intertidal macroalgae (Hovel et al., 2016). For example, in marine ecosystems, microorganisms such as epiphytic bacteria, fungi and archaea gather on the surface of algae, making this micro-habitat an extremely active interface between host and microbes (Wahl et al., 2012).

Long-term coexistence enables microorganisms to evolve adaptive characteristics closely relating to plants (Vorholt, 2012). Many epiphytes may have a mensal, commensal or parasitic relationships with plants, including macroalgae (Gopal and Gupta, 2016). Microbiota can help plants better absorb nutrients necessary for growth and development (Doty et al., 2016). Recently, the significant role of epiphytic microbiota in assisting plants maintain normal growth and metabolism has attracted scientists attention (Barott et al., 2011). Host-adapted microorganisms are more resistant to abiotic stresses such as harmful ultraviolet radiation (Kamo et al., 2018), oxidative stress and desiccation (Vorholt, 2012), and affect plant health by reducing biotic or abiotic stresses.

The surface of seaweed also provides habitat for microbial communities (Wiese et al., 2009). The continuous colonization of epiphytic microbiota on healthy algae indicates that there is a positive interaction between them and the host. For example, the growth and development of some green algae depend on specific bacteria (Provasoli and Pintner, 1980). Some microorganisms can provide vitamins (Droop, 2007), fatty acids (Karthick and Mohanraju, 2018), and other active substances to regulate the growth of algae. Furthermore, microorganisms can even produce antimicrobial agents for algae to enable the hosts to adapt to environmental stresses (Miranda et al., 2013; El Shafay et al., 2016). Studies have shown that their abundance is also related to availability of nutrients on the host surface (Paix et al., 2020).

Marine green macroalgae *Ulva* is widely distributed in coastal areas all over the world. It is commonly used as a model to study the environmental adaptability of biological life cycle (Juhmani et al., 2020). It has been reported that high nutrient content in seawater leads to an increase in the number of *Ulva*, which may lead to green tide (Zhang et al., 2019). The development of high-throughput sequencing technology makes it possible to characterize the composition of *Ulva* epiphytic microbiota. To our knowledge, the current research on the epiphytic microbiota of *Ulva* mainly focuses on the functional characteristics of microorganisms that responsible for *Ulva* morphogenesis and the different species composition that responsible for *Ulva* surface morphogenesis during the green tide (Nakanishi et al., 1996; Dobretsov and Qian, 2002; Spoerner et al., 2012; Ghaderiardakani et al., 2017, 2019; Weiss et al., 2017; Alsufyani et al., 2020; Chen et al., 2020; Gianmaria et al., 2020; Inaba et al., 2020; Juhmani et al., 2020; Liu et al., 2020; Qu et al., 2020). However, the biodiversity and functional relationship of epiphytic microbiota harbored by *Ulva* have not been fully explored. Previous studies have shown that bacterial communities located in different genotypes of the same algae usually have small differences. In addition to the influences of genotype, the composition of epiphytic bacterial community is also affected by seasons, environment and nutrients (El-Said and El-Sikaily, 2013).

Most studies show that α-Proteobacteria and γ-Proteobacteria are the main bacterial phyla colonizing the surface of algae (Bondoso et al., 2014). Different plants, such as rice (Edwards et al., 2015), grape (Zarraonaindia et al., 2015), sugarcane (De Souza et al., 2016), and citrus (Xu et al., 2018), have the same core microbiota (at the genus level), among which *Pseudomonas*, *Methylobacterium*, and *Sphingomonas* are the most common. This suggests that the core microbiome is selectively recruited and usually has the necessary functions to co-existence with the host. The identification methods and techniques of core microorganisms constitute the basis for studying how to construct a synthetic microbiota, revealing plant-microbiome interaction and possibly promoting plant growth.

*Ulva fasciata* are widely distributed in the rocky intertidal zone along the coast of China. Bacteria on algae vary with season and the life cycle of host (Lachnit et al., 2011). The purpose of this study was to determine (1) whether the epiphytic microbial community on the surface of *U. fasciata* varies at different growth stages, (2) whether the epiphytic microbiota is related to the growth stage of *U. fasciata*, and (3) whether the selected bacteria can promote the growth of *U. fasciata*.

The application of the bacterial treatment method in a large number of studies showed that the bacterial treatment method not only reveals how plants affect their microbial communities and how microbial communities affect plant growth and health but also has high operability and application value for agricultural production such as promoting plant growth and resisting plant diseases. It is believed that the application of the bacterial treatment method will also bring light to the solution of ecological problems such as green tide. Future studies will focus on the detailed identification of the nutrient fixation and promotion abilities of these isolates from the surface of *U. fasciata*.

## Materials and Methods

### Sampling and Determination of Environmental Parameters

**FIGURE 1:**
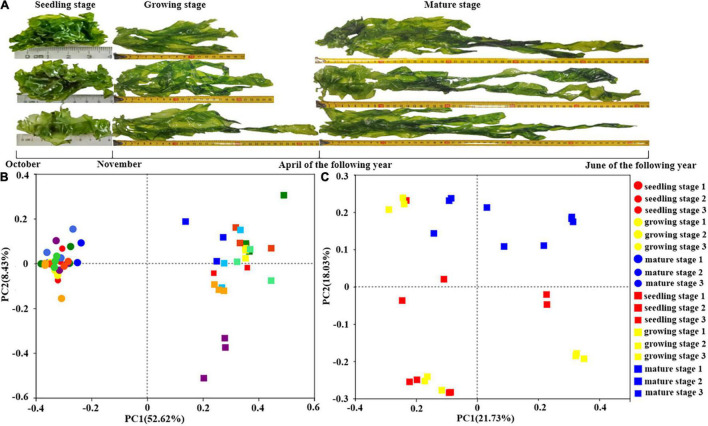
The surface microbiota of *Ulva fasciata* variation at different growth stages. **(A)** Photographs of *U. fasciata* samples in different life cycles **(B)** Principal Coordinate Analysis of microbiota changes on the surface of *U. fasciata* and seawater samples of different growth stages at Genus Level **(C)** Principal coordinate analysis of microbiota changes on the surface of *U. fasciata* in different growth stages at Genus Level (the circles represent the seawater sample and the squares represent the surface of the *U. fasciata* sample).

*Ulva fasciata* was randomly sampled from Nanao Island, Shantou, Guangdong, China (about 100 m from the coast) (116.53°E 23.11°N). In the seedling stage, *U. fasciata* were sampled once a week on November 9th, 16th, and 23th, that corresponding to seedling stages 1, 2, and 3, respectively. During the growth stage, *U. fasciata* were sampled on December 27, February 2, and March 28, which corresponding to growth stages 1, 2, and 3, respectively. At maturity, *U. fasciata* were collected on April 2, May 3, and June 3, corresponding to maturity stages 1, 2, and 3, respectively (Figure 1A). The samples were collected in three replicates, washed with sterile seawater, and stored in 50-mL sterilized centrifuge tubes.

During nine sampling, the *in situ* environmental parameters (temperature, salinity, pH, and dissolved oxygen concentration) were recorded (Supplementary Table 1). At the same time, 1 L seawater samples were collected, filter through 0.45 and 0.22 μm membranes to remove impurities and collect bacteria. The filtrates (0.22 μm filter membrane) were stored in sterile sampling bags and the filtered water was cryopreserved for the determination of physical and chemical parameters, including dissolved organic carbon (TOC), inorganic carbon (TIC), total nitrogen (TN), and total carbon (TC) according to the methods described by Urgun-Demirtas et al. (2008). All samples were placed in a foam box with ice cubes and transferred to the laboratory within 4 h. In the laboratory, the microbiota harboring the surface of *U. fasciata* was then immediately collected. The *U. fasciata* samples in a 50-mL sterilized centrifuge tube was mixed with an appropriate amount of sterile seawater and treated with ultrasonic (1 min, 50 W, 5s/5s). Algae were removed, and then the suspension was centrifuged at 4,000 × *g* for 10 min to collect the pallet, which was then used for bacterial DNA extraction.

### DNA Extracting and 16S rDNA Sequencing

Bacterial DNA was extracted using the CTAB method (Zhou et al., 1996). DNA quality and integrity were evaluated by gel electrophoresis. Samples with high-quality DNA were stored at −80°C until further use. The DNA of all samples was sent to Shanghai Majorbio Bio-Pharm Technology Company for sequencing and data analyses.

### Sequence Data and Statistical Analysis

To determine the species and abundance of bacteria, the V3–V4 region of the 16S rRNA gene was amplified using primers 806R (5′-GGACTACHVGGGTWTCTAAT-3′) and 338F (5′-ACTCCTACGGGAGGCAGCAG-3′), and the PCR products were sequenced using Illumina MiSeq sequencing platform. The sequencing data were stitched by Flash V1.2.11, and then the optimized Fastq files were obtained after quality control by Fastp V0.19.6. The sequences were preprocess using the software Usearch 7 (parameter setting: minsize 2), and the sequences were grouped into operational classification units (OTUs) for species level classification using 97% sequence similarity. After obtaining the OTU classification table, those OTUs assigned to chloroplasts or mitochondria and eukaryotes were excluded from the data set. OTU counts were normalized, the original count was divided by the total number of each sample, and then multiplied by the median to calculate the relative abundance. Using QIIME V1.9.1, the taxonomic information was assigned to each representative OTU sequence and filtered OTUs were used to calculate alpha diversity indexes, including Shannon, Chao1, and Simpson. Calculate the Bray-Curtis distance for all pairs of samples to determine the amount of diversity shared between communities (β-diversity) (Liu et al., 2021). Principal Co-ordinates Analysis (PCoA) sorting was performed according to Bray-Curtis distance. Adonis based on Bray distance was used to calculated the significant difference between bacterial community and the null hypothesis (Anderson, 2001). Dispersion of microbial communities was evaluated using the pairwise permutation tests with Tukey’s HSD. The Kruskal–Wallis test was used to determine whether there were significant differences in the abundance of bacterial classes. Bacterial taxonomic biomarkers for bacterial classification were obtained by referring to the random forest algorithm (Zhang et al., 2018). The Cytoscape software package was used for visualization. All of the above software used default parameters.

### Construction of Bacterial Treatment and *U. fasciata* (WSB) Growth Promotion Test and Plant Growth Promotion Experiment

Samples of *U. fasciata* were collected at different times, rinsed and immediately transferred to the laboratory. The blade surface was gently scraped with a sterile knife, and the scraped materials were gradually diluted and spread on the marine agar plate 2216E, and placed in an incubator at 25°C for 3–4 days. Single colonies were selected, streaked, purified, and inoculated in marine broth 2216E. The 16S rRNA gene of bacteria was amplified with universal primers 27F and 1492R, and the samples were sent to Bioengineering (Shanghai) for sequencing. The phylogenetic names of the isolates were identified by EzBioCloud database. A total of 228 pure strains were obtained. These strains were cultured on marine broth 2216E to logarithmic growth phase. The bacterial suspension was mixed with 40% sterilized glycerol in the ratio of 1:1. These strains were selected as candidate strains for bacterial treatment. To test the promoting effect of bacterial treatment on plant growth, *U. fasciata* without surface bacteria (WSB) was cultured with isolates.

*Ulva fasciata* without surface bacteria was prepared as follow:

The surface of *U. fasciata* was washed with sterile seawater and ultrasonic waves (1 min, 50 KHz, 5s/5s) for three times to remove bacteria. The mixture of antibiotics (including 100 μg/mL streptomycin sulfate, 0.25 μg/mL amphotericin, 100 μg/mL ampicillin, and 0.1 μg/mL imidazole) was added to the culture tube and incubated for 2 days; the same dose of antibiotics was added on the third day. The surface of *U. fasciata* was quickly scrubbed with a sterile cotton swab dipped in a multi-enzyme cleaning solution (3M), and then rinsed with sterile seawater. The obtained SBR was evaluated through three methods: (1) PCR detection of seawater collected from the last rinsing of *U. fasciata* and algal abrasive solution in the range of about 1500 bp using primers 27F and 1492R, (2) the seawater collected from the last time rinsing of *U. fasciata* was used to culture on marine agar 2216E to confirm the growth of bacteria in 7 days, (3) the sterile surface of *U. fasciata* was stained with 1% (v/v) of DAPI for 20 min, the excess DAPI was removed with sterile water, and then the confocal laser scanning microscopy (CLSM) was used to check whether there were bright blue bacterial spots (bacteria) in the sample. Samples were imaged with CLSM (LSM 700, Zeiss, Germany) and x100 oil immersion objective. Select DAPI channel 400–630 nm. Four random locations were scanned on each sample.

The experiment consisted of three bacterial treatment groups, sterile seawater (C group), *Bacillus cereus* U5-30 (P group) and *Hyunsoonleella* sp. HU1-3 (H group). The bacteria were incubated in marine broth 2216E at 25°C for 2 days, centrifuged, washed twice with sterile seawater, and then resuspended to a density of 10^7^ cells per mL. The volume of bacterial liquid added to each bacterial treatment was 5 mL. The mixed bacteria were added to a bottle connected to filtered air and contained 800 mL of sterile seawater, and then co-cultured with *U. fasciata* (WSB) for 7 days. At the end of the experiment, the co-cultured *U. fasciata* was washed with sterile seawater, and the surface bacterial strains of *U. fasciata* were obtained by ultrasonic shock. The 2216E marine agar plate was used to test whether the bacterial treatment strains survived after 7 days.

To determine the growth rate, at the beginning of the co-culture experiment, young *U. fasciata* with similar growth trend and size were selected, weighed (set up as W_t_) and sterilized. The vase was placed in a light incubator for 7 days (temperature, light intensity, photoperiod and air supply time were 20°C, 4000 LX, 12 h:12 h, and 24 h, respectively). After 7 days of co-culture, *U. fasciata* was taken out, the surface seawater was removed and weighed (W_0_). Growth rate was calculated by the formula of [(W_t_/W_0_)^1/t^-1] × 100% (t is days of culture) (Yong et al., 2013). During the experiment, each bacterial treatment group was guaranteed at least three biological replicates. The contents of soluble protein, soluble sugar, phycocyanin, and chlorophyll-a (Schwenzfeier et al., 2011) were determined to characterize the biomass of *U. fasciata.*

The bacteria strains were incubated in 2216E liquid medium at 25°C for 3 days, with or without 100 μg/mL of L-tryptophan, and then centrifuged. After that, the supernatant was diluted to OD600 = 0.8 with sterile seawater, and 10 mL of culture were added to a bottle containing 800 mL of sterile seawater with *U. fasciata* (WSB) and inoculated for 7 days. In the experiments, six supernatant were used, including sterile seawater (A group), 2216E liquid medium (B group), supernatant of *B. cereus* strain U5-30 living in 2216E liquid medium (C group), supernatant of *Hyunsoonleella* sp. strain HU1-3 living in 2216E liquid medium (D group), supernatant of *B. cereus* strain U5-30 living in 2216E liquid medium and L-tryptophan (E group), supernatant of *Hyunsoonleella* sp. strain HU1-3 living in 2216E and L-tryptophan (F group). Six groups of supernatants were separately cocultivated with *U. fasciata* (WSB), and the growth rate of *U. fasciata* was measured by the same method as above.

### Genome Sequencing and Assembly

In order to illustrate how *Hyunsoonleella* strain HU1-3 promotes the growth of *U. fasciata*, the extraction of its genome was sent to BGI Corporation for genome test, and double-terminal sequencing was performed based on Illumina PE150 platform. Raw data is processed by low-quality filtering. Low quality bases of more than 40 bp were removed (quality value 38 or less), N bases were up to 10 bp, and the overlap between junctions was more than 15 bp. The repeated contamination is then removed to obtain valid data. The genome size was estimated by K-mer statistical analysis, and the raw sequencing reads were trimmed and assembled by SOAPdenovo v2.04 software^1^. The FastQC software was used to analyze sequence quality (Brown et al., 2017). GeneMark software (V.4.17) (Besemer et al., 2001) was used for comparison and genomic protein-coding genes were obtained. These gene sequences were compared with the known Nr database (non-redundant protein database) (Li et al., 2002). For the comparison results of each sequence, the highest score (identity ≥40%, coverage ≥40%) was selected for gene function annotation.

### Strains Indole-3-Acetic Acid Production and Solubilization of Phosphate

The bacteria strains were incubated in 2216E liquid medium at 25°C for 3 days. 1-mL incubation fluid was centrifuged, the cell precipitates were washed twice with PBS, and then resuspended to 107 cells/mL. 1 mL of the suspension was mixed with 10 mL of liquid medium (with and without 100 μg/mL of tryptophan). After 3 days, 50 μL of supernatant was collected and mixed with 50 μL of Salkowski reagent (50 mL 35% HClO_4_ + 1 mL 0.5 mol/L FeCl_3_). After 25 min, the absorbance value was read at 530 nm (Bric et al., 1991) to measure the production of bacterial IAA. Bacteria were inoculated in 50 mL sterilized liquid NBRIP medium at a dose of 0.2% and cultured in a flask at 25°C for 7 days. The culture was centrifuged at 5,000 RPM for 20 min, and the precipitation of P_2_O_5_ was determined by molybdenum blue method according to Galhardo and Masini (2000) to measure the solubility of phosphate. All measurements were conducted in triplicate.

## Results

### Succession of *U. fasciata* Surface Bacterial Communities and Model of Correlation Between Surface Bacterial Taxonomic Biomarkers

In order to investigate the variations of microbes harboring the surface of *U. fasciata* under natural conditions, seawater samples and *U. fasciata* samples, a total of 2,969,125 high-quality sequences were obtained from all samples. The bacterial community structure over time in different samples is illustrated in Figures 1B,C. There was a significant difference (*P* < 0.01) between the microbial community living in seawater and the *U. fasciata* surface community (Figure 1B). PCoA analysis also showed that there were different microbiota harboring the surface of *U. fasciata* at different growth stages (*R*^2^ = 0.68, *P* < 0.01, adonis) (Figure 1C). This indicated that the growth stage plays a significant role in the establishment of microbiota inhabiting the surface of *U. fasciata.* Mature stage samples were close to one another, while seedling stage samples were relatively far away from one another (Supplementary Figure 1). The microbial community in mature stage overlapped with that in seedling stage and growth stage, indicating that the microbial community is in a transitional state between mature and seedling/growth stages. Similar results can be obtained by calculating the community diversity index (Shannon) and community richness index (Chao and ace) (Table 1), in which the alpha-diversity index of surface bacteria was significantly lower than that of seawater bacteria, while the diversity index of *U. fasciata* surface bacteria remained relatively stable.

**TABLE 1:** Microbiodiversity indices of *U. fasciata* surface and seawater at different growth stages.

Sample	Stage	Shannon	Ace	Chao
Seawater	Seedling stage 1	4.15 ± 0.30	2153.21 ± 438.36	1122.85 ± 126.96
	Seedling stage 2	3.67 ± 0.50	1185.38 ± 466.28	711.35 ± 204.09
	Seedling stage 3	4.31 ± 0.36	1880.29 ± 212.66	1115.01 ± 116.76
	Growing stage 1	4.25 ± 0.07	2245.17 ± 225.93	1252.29 ± 161.64
	Growing stage 2	4.14 ± 0.11	1774.78 ± 383.26	986.01 ± 141.13
	Growing stage 3	4.20 ± 0.29	2151.60 ± 384.07	1280.32 ± 109.10
	Mature stage 1	3.88 ± 0.65	1948.03 ± 930.45	1110.24 ± 328.99
	Mature stage 2	4.23 ± 0.23	1826.69 ± 613.97	1161.50 ± 300.89
	Mature stage 3	4.22 ± 0.14	2082.02 ± 487.13	1129.71 ± 173.52
*Ulva*	Seedling stage 1	4.30 ± 0.12	463.64 ± 210.61	436.64 ± 143.87
	Seedling stage 2	3.88 ± 0.15	332.09 ± 139.52	292.25 ± 67.94
	Seedling stage 3	3.07 ± 1.69	337.85 ± 212.87	274.22 ± 138.34
	Growing stage 1	4.22 ± 0.10	565.49 ± 137.80	466.52 ± 41.52
	Growing stage 2	4.48 ± 0.09	477.85 ± 90.53	422.89 ± 68.10
	growing stage 3	4.51 ± 0.18	541.79 ± 80.81	522.55 ± 91.61
	Mature stage 1	4.02 ± 0.44	396.08 ± 103.21	394.94 ± 118.55
	Mature stage 2	4.18 ± 0.13	734.79 ± 167.47	574.22 ± 87.80
	Mature stage 3	4.40 ± 0.05	792.73 ± 149.49	574.09 ± 66.85

The relative abundance of epiphytic microbiota of *U. fasciata* and seawater is illustrated in Supplementary Figures 2A,B. Seawater samples were dominated by marine bacteria, such as HIMB11 and NS5 marine groups (Supplementary Figure 2A). Microbial community abundance were significant differences in the surface of *U. fasciata* with the seawater (Supplementary Figure 2B). The relative abundance of Proteobacteria and Bacteroidetes on *U. fasciata* surface was significantly higher that in seawater. The results of Kruskal-Wallis analysis (Supplementary Figure 3A) showed that although the relative abundance of these species increased or decreased throughout the growth period, there was no significant difference. Actinobacteria and Firmicutes changed greatly throughout the growth stage. At the genus level, there were significant differences in the relative abundance of some species (Supplementary Figure 2C). For instance, at the seedling stage, the relative abundances of Rhodobacteraceae (13.0%, *P* < 0.01, Kruskal–Wallis), *Acinetobacter* (6.2%, *P* < 0.01, Kruskal–Wallis), and *Vibrio* (1.2%, *P* < 0.01, Kruskal–Wallis) was significantly lower, while the relative abundance of *Ralstonia* (2.3%, *P* < 0.01, Kruskal–Wallis), *Erythrobacter* (2.8%, *P* < 0.01, Kruskal–Wallis), and *Rhodococcus* (2.4%, *P* < 0.01, Kruskal–Wallis) were significantly higher. The composition of microbiota on the surface of *U. fasciata* varied greatly throughout the growth, but the dominant species of microbiota were Rhodobacteraceae, *Ralstonia, Erythrobacter, Rhodococcus, Acinetobacter, Alteromonas, Vibrio, Halomonas*, and *Granulosicoccus.*

The regression analysis between the relative abundance of bacteria on the surface of *U. fasciata* species and the growth stage was established and revealed that the model explained 56% of the surface of *U. fasciata* microbiota variance related to *U. fasciata* growth stage (Supplementary Figure 4). At the class level, the class number (*n* = 30) was stable and relative to the cross-validation error curve. Therefore, these 30 bacterial classes, especially *Rhodococcus* and *Hyunsoonleella*, were defined as biomarker groups in the model.

### Effects of Environmental Factors and Physiological Parameters of *U. fasciata* on Bacterial Community

**FIGURE 2:**
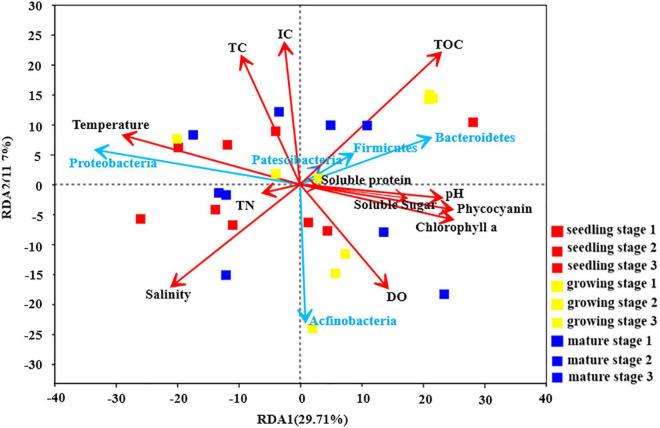
Redundancy Analysis of *U. fasciata* surface microorganisms with environmental factors and plant physiological indexes at *Phylum* level (The red arrows represented different environmental parameters and physiological indicators of *U. fasciata* and the blue arrows represented the top five groups of bacteria in abundance).

The relationship between environmental factors and the abundance of different phyla is shown in Figure 2. At the phylum level, the relative abundance of Patescibacteria, Firmicutes and Bacteroidetes was positively correlated with the physiological parameters of different carbon forms. As mentioned above, the *U. fasciata* growth stage is the best explanation for the variation of microbial community. However, the environmental and physiological parameters of *U. fasciata* at different growth stages have different explanations for the variation of microbial community (Table 2). Dissolved oxygen (DO) and Phycocyanin had no effect on the microbial community on the surface of *U. fasciata* (*R*^2^ = 0.04 and 0.05, respectively, *P* > 0.05, Adonis). TOC, pH, soluble protein and TN had moderate effects on the microbial community on the surface of *U. fasciata*. IC and TC had the greatest effect on the microbial community on the surface of *U. fasciata* (*R*^2^ = 0.13, 0.14, respectively, *P* < 0.05, Adonis). The results showed that environmental factors and physiological parameters of *U. fasciata* also affected the structure of surface bacteria.

**TABLE 2:** Effects of growth stage, environmental factors, and physiological parameters of *U. fasciata* on *U. fasciata* surface microbiota.

Characteristics	Sums of sqs	Mean sqs	*F* model	*R* ^2^	*P*-value	*P*.adjust
Growth stages	4.11	0.51	4.82	0.68	0.001	0.004
IC (mg/L)	0.81	0.81	3.88	0.13	0.001	0.004
TC (mg/L)	0.83	0.83	3.99	0.14	0.001	0.004
TOC (mg/L)	0.60	0.60	2.76	0.10	0.003	0.009
pH	0.57	0.57	2.62	0.10	0.004	0.009
Soluble protein (mg/g)	0.64	0.64	2.95	0.11	0.004	0.009
TN (mg/L)	0.59	0.59	2.69	0.10	0.005	0.009
Salinity (‰)	0.48	0.48	2.15	0.08	0.014	0.023
Soluble sugar (mg/g)	0.44	0.44	1.99	0.07	0.025	0.036
Chlorophyll A (mg/g)	0.44	0.44	1.99	0.07	0.038	0.049
Temperature (°C)	0.36	0.36	1.57	0.06	0.108	0.128
Phycocyanin (mg/g)	0.30	0.30	1.32	0.05	0.217	0.235
DO(ppm)	0.27	0.27	1.17	0.04	0.319	0.319

*The first column of the table represents grouping factors or environmental factors. R^2^ represents the degree of explanation of grouping factors to sample differences. The larger R^2^ is, the higher the degree of explanation of grouping factors to sample differences. P-value less than 0.05 indicates high reliability of this test.*

### *Hyunsoonleella* sp. HU1-3 Strains Promote Plant Growth

A total of 228 bacterial strains were isolated from the surface of *U. fasciata* at different growth stages (Supplementary Figure 5). These strains were classified into three phyla, including Firmicutes, Bacteroidetes, and Proteobacteria. Although bacteria were still present in the *U. fasciata* (WSB) lapped samples (Supplementary Figure 6C) and intercellular bacteria were detected by CLSM (Supplementary Figure 6P), the quality of *U. fasciata* (WSB) was comparable to that of wild *U. fasciata* (Supplementary Figure 7) (*P* > 0.05, ANOVA).

The prediction results of machine forest model suggest that members of Rhodobacteraceae and Flavobacteriaceae may play an important role in the growth of *U. fasciata*. Therefore, bacterial treatment experiments were carried out to investigate the growth-promoting effect of Rhodobacteraceae and Flavobacteriaceae on *U. fasciata*. The role of endophytic bacteria was ignored in the experiment bacterial treatments. Among the isolates, *Hyunsoonleella* sp. HU1-3 demonstrated the greatest growth promoting potential on *U. fasciata*. Compared with other bacteria of *Hyunsoonleella*, its 16S rRNA gene sequence similarity and average nucleotide identity values were less than 98.7 and 95%, respectively (Chun et al., 2018), so it is considered as a new species of the genus.

**FIGURE 3:**
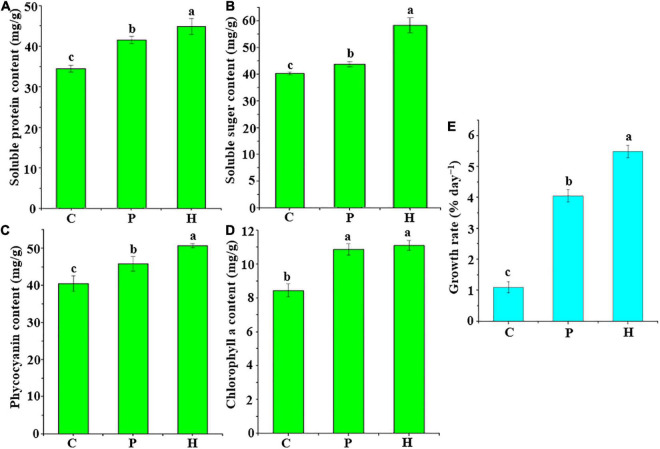
Changes of growth rate and four physiological indices of bacterial treatment and *U. fasciata* (WSB). **(A)** Soluble protein content, **(B)** Soluble sugar content, **(C)** Phycocyanin content, **(D)** Chlorophyll *a* content, and **(E)** Growth rate. C group used sterile seawater for the negative control, P group only contains *Bacillus cereus* U5-30 strain was used to be the positive control, H group contained only one strain of *Hyunsoonleella* sp. HU1-3, the difference coefficient represents the difference between C, P, and H group.

The four physiological indices and growth rate of *U. fasciata* of group H [co-culture of *U. fasciata* (WSB) and *Hyunsoonleella* sp. HU1-3] were significantly higher than those of group C (only seawater was added as the negative control) and group P [co-culture of *U. fasciata* (WSB) and *B. cereus* U5-30 as the positive control] (*P* < 0.05, ANOVA) (Figure 3). In fact, compared with group P, the positive effect of *Hyunsoonleella* HU1-3 on soluble protein, soluble sugar and phycocyanin of *U. fasciata* showed that the strain significantly increased the biomass of sterile *U. fasciata* (*P* < 0.05, ANOVA) (Figures 3A–D). The growth rate of group H (5.49% day^–1^) and group P (4.05% day^–1^) was significantly higher than that of group C (1.10% day^–1^) (Figure 3E).

**FIGURE 4:**
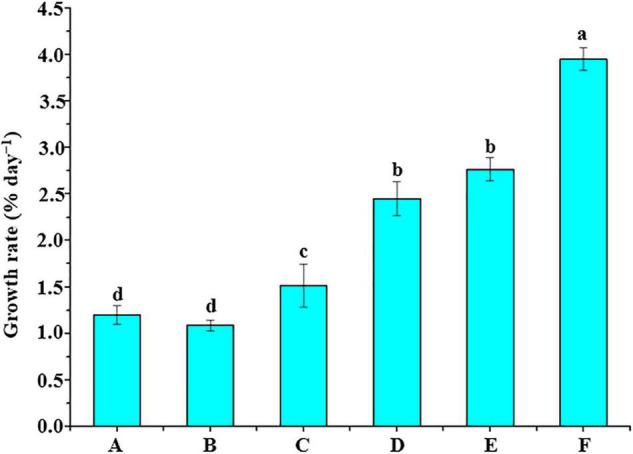
Supernatant of bacterial culture and *U. fasciata* (WSB) growth promotion test. A group added sterile seawater, B group added sterile 2216E liquid medium, C group added the supernatant of the strain *B. cereus* U5-30 living in 2216E, D group added the supernatant of the strain *Hyunsoonleella* sp. HU1-3 living in 2216E, E group added the supernatant of the strain *B. cereus* U5-30 living in 2216E + L-tryptophan, F group added the supernatant of the strain *Hyunsoonleella* sp. HU1-3 living in 2216E + L-tryptophan. The difference coefficient represents the difference between A and F group.

Six different groups of bacterial supernatants were cocultured with *U. fasciata* (WSB) (Figure 4). There was no significant difference in the growth rate of *U. fasciata* between group A (sterile seawater) and group B (sterile 2216E liquid medium) (*P* > 0.05, ANOVA). Interestingly, the supernatant of group C (*B. cereus* strain U5-30) and group D (*Hyunsoonleella* sp. HU1-3) significantly increased the growth rate of *U. fasciata* (1.52% day^–1^ and 2.45% day^–1^, respectively; *P* < 0.05, ANOVA). Surprisingly, the *U. fasciata* growth rate of group E and group F (2.77% day^–1^ and 3.95% day^–1^, respectively; *P* < 0.05, ANOVA) was significantly higher than that of group C.

### Genetic Elements Involved in *Hyunsoonleella* sp. HU1-3 Plant Growth Promotion

**FIGURE 5:**
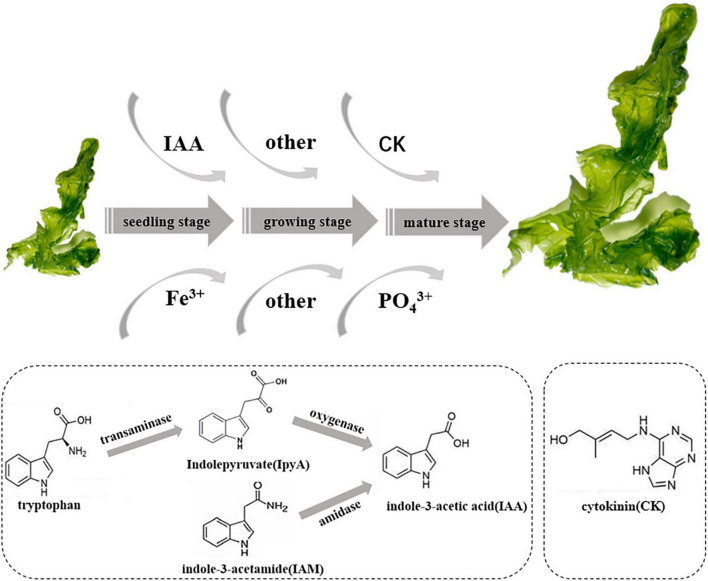
Schematic diagram of potential promoting growth of *U. fasciata* of strain *Hyunsoonleella* sp. HU1-3.

Based on the genome of *Hyunsoonleella* HU1-3 (Figure 5 and Supplementary Table 2), several amidases (such as N-acetylmuramoyl-L-alanine amidase LytC and 2-oxoglutaramate amidase) and tryptophan related enzymes (such as tryptophan 2,3-dioxygenase and tryptophan-tRNA ligase) were identified, indicating that *Hyunsoonleella* sp. HU1-3 may produce IAA through IpyA and IAM pathways. Genes involved in the synthesis of cytokinins (CK) have also been annotated, such as tRNA-dimethylallyltransferase, tRNA-2-methylthio-N6-dimethylallyladenosine, and cytokinin-nucleoside 5-monophosphate ribose hydrolase. These results indicate that *Hyunsoonleella* sp. HU1-3 has the potential to synthesize a variety of CKs. Furthermore, seven phosphate synthases genes were found in the genome of *Hyunsoonleella* sp. HU1-3. Interestingly, *Hyunsoonleella* sp. HU1-3 contains genes involved in vitamin synthesis (thiamine-phosphate synthase and thiamine-monophosphate kinase). Other genes annotated as iron carrier enzymes (such as iron-sulfur cluster carrier protein and iron-dependent repressor IdeR) were also found in the genome.

### Indole-3-Acetic Acid Production and Phosphate Solubility Capacity of Strain

Plant growth is known to depends on several transaminase proteins, especially growth hormones, such as IAA. In the absence of tryptophan, the IAA yield of *B. cereus* U5-30 was 209.70 ± 3.64 μg/mL. The addition of tryptophan increased the IAA production to 375.16 ± 3.59 μg/mL. The phosphate solubility of *B. cereus* U5-30 was 191.18 ± 2.87 μg/mL. On the other hand, the IAA yield and phosphate solubility of *Hyunsoonleella* sp. HU1-3 without tryptophan were 227.64 ± 0.82 μg/mL, and 213.98 ± 1.15 μg/mL, respectively. It is speculated that the IAA yield and phosphate solubility of *Hyunsoonleella* sp. HU1-3 are better than *B. cereus* U5-30 (Table 3).

**TABLE 3:** The production of IAA and the solubilization capacity of phosphate in the strain.

Stains	Without tryptophan (μg/ml)	With tryptophan (μg/ml)	Soluble phosphate (μg/ml)
*Bacillus cereus* U5-30	209.70 ± 3.64	375.16 ± 3.59	191.18 ± 2.87
*Hyunsoonleella* sp. HU1-3	227.64 ± 0.82	395.95 ± 3.11	213.98 ± 1.15

## Discussion

Epiphyte studies during plant growth are usually carried out under greenhouse conditions (Edwards et al., 2015). We conducted field sampling to represent the life cycle of *U. fasciata. U. fasciata*’s growth slowly in November (seedling stage) and reaches maturity in April of the following year. In June, with the increase of seawater temperature, the *U. fasciata* will bleached, rotten and immediately washed away by seawater, which makes sampling difficult. Therefore, we only focus on *U. fasciata* that grows well from seedling stage to decay stage. Roth-Schulze et al. (2018) demonstrated that the bacterial diversity on the surface of *U. fasciata* is different from the surrounding seawater. The PCoA analysis in this study revealed that the composition of epiphytic microbiota of *U. fasciata* was significantly different from that of surrounding seawater. In a previous study, Lachnit et al. (2011) reported that the microbial composition on the surface of *Ulva infantis* varied with the growth stage. Similarly, it was reported that the dynamic change of surface microbiota through the plant life cycle depends on the growth stage of plant (Chaparro et al., 2014; Dombrowski et al., 2016). The dominant bacteria on the surface of *U. fasciata* were Proteobacteria and Bacteroidota. This finding is consistent with Burke et al. (2011).

The core microbiome is defined as a group of shared members in microbial communities from similar habitats (Shade and Handelsman, 2012). Thus, the discovery of core microbiome is very important to understand the stable components of complex microbial assemblages. In this study, the core microbiome was consistent at different growth stages. Some Rhodobacteraceae, *Ralstonia*, *Erythrobacter*, *Rhodococcus*, *Acinetobacter*, *Alteromonas*, *Vibrio*, *Halomonas*, and *Granulosicoccus* were the dominant genera on the surface of *U. fasciata*. It is reported that *Ulva australis* are mainly composed of Alphaproteobacteria, Bacteroidetes, Planctomycetes, and unclassified Gammaproteobacteria (Burke et al., 2011), while the bacterial community associated with green seaweeds is mainly composed of Proteobacteria, Bacteroidetes, Verrucomicrobia, Cyanobacteria, Planctomycetes, Actinobacteria, and Firmicutes (Selvarajan et al., 2019). The species of core microbial communities on *Ulva* surface were similar. However, the differences of green algae epiphytic communities in different environments may be explained by compounds secreted by green algae surface that regulate the composition of microbial communities.

In addition, most of the genera level biomarker groups showed high relative abundance at the corresponding growth stages (e.g., Rhodobacteraceae and Flavobacteriaceae). It is reported that Flavobacteriaceae bacteria are beneficial to the growth of *Ulva*. For example, Spoerner et al. (2012) reported that the addition of MS6 from *Maribacter* sp. strain restored the morphogenesis of *Ulva mutabilis*. MS6 is similar to auxin it induces cell wall formation and promotes the growth of basal stem cells. Many other studies also have shown that Rhodobacteraceae promote plant growth (Stevens et al., 2017; Abraham and Silambarasan, 2018; Kuhl et al., 2019; Vergani et al., 2019).

Ambient environmental parameters are another important factor affecting the abundance and diversity of microbial community on the surface of *U. fasciata*. McDonough and Watmough (2015) reported that epiphytic microbiota has a better positive correlation with environmental factors, such as temperature and pH. The authors also emphasized that the of the specificity, abundance and diversity indices of epiphytes were negatively correlated with the concentration of ambient nutrients (McDonough and Watmough, 2015). Moreover, Stratil et al. (2014) revealed that temperature, salinity and DO are the main environmental factors affecting the structure of microbial community. However, these findings contradicted our results and those of Pei et al. (2021), which showed that temperature, salinity and DO have no significantly effects on algal epiphytic microbiota. In this study, IC and TC had a great influence on the surface microbiota of *U. fasciata*. It also reported that algae are more associated with nutrients in the surrounding environment (Aires et al., 2016). Therefore, the authors conclude that the species of algal epiphytic bacteria in different environments are the result the synergistic effects of sampling methods, growth indexes of algae and environmental factors (Selvarajan et al., 2019).

The results of this study revealed that the growth promoting effect of strain HU1-3 (belonging to Flavobacteriaceae) was the best on *U. fasciata*. *B. cereus* U5-30 has the closest genetic relationship with *Bacillus cereus* AR156, with 100% 16S rRNA gene sequence similarity. *B. cereus* AR156 was isolated from soil and is reported to promote plant growth (Jiang et al., 2017). In this study, *B. cereus* U5-30 can well promote the growth of *U. fasciata*, so it can be used as a positive control of bacterial treatment experiment. Compared with *B. cereus* U5-30, the biomass, phycocyanin, soluble sugar, soluble protein and growth rate of *U. fasciata* were greatly improved after co-culture with *Hyunsoonleella*, indicating that *Hyunsoonleella* sp. HU1-3 was a better growth promoter for *U. fasciata*. To our knowledge, this is the first study of *Hyunsoonleella* sp. as a growth promoter in plants.

The co-culture of different bacterial supernatants with *U. fasciata* (WSB) also indicated that *Hyunsoonleella* sp. HU1-3 increased the growth rate of *U. fasciata*. The genomic sequencing of *Hyunsoonleella* sp. HU1-3 revealed that the strain contained auxin related genes (an important phytohormone for the plant growth), involved in the production of IAA and CK, and several other genes encoding different enzymes (phosphate synthases genes and iron carrier enzymes genes). Plant growth depends on specific growth hormones, such as IAA and CK. These endogenous compounds present in plant tissues are considered to be signals to coordinate plant development (Kudo et al., 2010). This is consistent with previous reports that many Flavobacteriaceae produce auxin and play an important role in promoting plant growth (Elena et al., 2007; Kaul et al., 2018; Vukanti, 2020; Kalyanasundaram et al., 2021). Various phosphatases in the strain not only improve the phosphorus absorption capacity of plant, but also coordinate the transformation of insoluble, inorganic and organic forms of phosphorus into bioavailable forms of phosphate in the growing environment. Soluble phosphorus microorganisms can also improve the efficiency of nitrogen fixation, accelerate the accessibility of other trace elements, and promote the synthesis of iron chelating compounds (Kour et al., 2019). It is reported that inoculating different crops such as corn, tomato and mung bean with phosphorus-solubilizing bacteria can promote plant growth (Sandhya et al., 2010). The presence of genes encoding iron carrier and phosphatase further demonstrated that *Hyunsoonleella* sp. HU1-3 has the potential function of promoting the growth of *U. fasciata.* Further studies are highly recommended in the near future to determine which substances contribute to the growth of *U. fasciata* biomass.

Although *U. fasciata* grows in seawater, the microbial community on the surface of *U. fasciata* seems to be some specific algae-related communities, which are essentially different from the microbial community in the surrounding seawater (at the phylum level). This may be caused by different living environments, such as the nutrient concentration in seawater is relatively low, while the surface of algae emits organic carbon and nutrients (Pregnall, 1983). Previous studies have shown that although the community is variable, there is always a core population on the surface of the algae (Tujula et al., 2010). The results of this study also confirmed that the same bacteria were detected on the surface of *U. fasciata* samples at different growth stages, indicating that these bacteria can be colonized on the surface of algae as a core community. Besides, there were significant differences in epiphytic bacterial community in different *U. fasciata* samples, indicating the functional redundancy of *U. fasciata* epiphytic bacterial communities. This conclusion is consistent with the redundancy hypothesis (Naeem, 1998), which assumes that more than one species can play a specific role in an ecosystem, so as to endow the ecosystem with a certain degree of anti-disturbance ability. This hypothesis has also been confirmed to be applicable to laboratory microbial communities (Leflaive et al., 2008) and soil microbial communities (Yin et al., 2000; Persiani et al., 2008). Although functional redundancy can explain the variability of the epiphytic bacterial community of *U. fasciata*, it cannot explain the significant difference between this algae-associated community and the planktonic bacterial community living in the surrounding seawater. This suggests that there may be a selective mechanism that determines the environment in which bacterial communities exist.

The lottery hypothesis asserts that species with similar nutritional abilities will randomly replenish the ecosystem and occupy space (Sale, 1976). This hypothesis was proposed to explain the coexistence of corals and fish occupying the same ecological niche, but it is also consistent with the observations described in this study. It is assumed that various bacteria in algae-associated communities have the necessary metabolic capacity and colonize in the niches on the surface of algae. Due to functional redundancy, any bacterial species in the community will first colonized when they happen to encounter and occupy the surface of algae. This can explain the community variability between different *U. fasciata* samples. Bacterial species from the surface of the *U. fasciata* may not be able to adapt to plankton habitat in seawater, which is why the bacterial species found on the surface of *U. fasciata* are rarely detected in the surrounding seawater. The lottery hypothesis was conceived for dozens of species of fish that living in corals. It will be interesting to study whether this hypothesis also applies to communities containing thousands of bacterial species. It will also be of great interest to determine whether the isolated bacterial community is unique to the *U. fasciata* or is simply indicative of a surface-associated lifestyle.

## Conclusion

In conclusion, a new bacterial species, *Hyunsoonleella* sp. HU1-3 was isolated from the surface of *U. fasciata.* This isolate significantly promoted the growth of *U. fasciata*. Genomic analysis revealed that the isolate process genes encoding the synthesis of growth hormones, phosphate synthases genes vitamins and iron carrier enzymes. The combination of high-throughput sequencing and bacterial treatment approaches helps to reveal the interaction between plants and microorganisms under the condition of reducing unknown variables.

## Data Availability Statement

The datasets presented in this study can be found in online repositories. The names of the repository/repositories and accession number(s) can be found below: https://www.ncbi.nlm.nih.gov/genbank/, MW527409; https://www.ncbi.nlm.nih.gov/genbank/, JAEPJQ000000000; https://www.ncbi.nlm.nih.gov/genbank/, PRJNA766413.

## Author Contributions

HW and ZH designed the study, wrote the manuscript, and contributed to the data analysis. AE, YL, WL, QC, YX, and TP performed the experiments and data acquisition. HW, TP, YL, WL, and ZH contributed to the treatment of the raw data. All authors contributed to the article and approved the submitted version.

## Conflict of Interest

The authors declare that the research was conducted in the absence of any commercial or financial relationships that could be construed as a potential conflict of interest.

## Publisher’s Note

All claims expressed in this article are solely those of the authors and do not necessarily represent those of their affiliated organizations, or those of the publisher, the editors and the reviewers. Any product that may be evaluated in this article, or claim that may be made by its manufacturer, is not guaranteed or endorsed by the publisher.

## Footnotes

1
http://soapdenovo2.sourceforge.net/
