# Supplementary material for: Hyunsoonleella sp. HU1-3 Increased the Biomass of Ulva fasciata
Source: Front Microbiol. 2022 Jan 31;12:788709. doi: 10.3389/fmicb.2021.788709 (PMC8841488; doi:10.3389/fmicb.2021.788709)
Supplement: Supplementary file 1 [file Data_Sheet_1.pdf]

Hyunsoonleella sp. HU1-3 increased the biomass of *Ulva fasciata*

Han Wang[1#], Ali Mohamed Elyamine[1#], Yuchun Liu[1], Wei Liu[1], Qixuan Chen[1], Yan Xu[1,2], Tao Peng[1], Zhong Hu[1,3*]

[1]Key Laboratory of Resources and Environmental Microbiology, Department of Biology, Shantou University, Shantou, Guangdong 515063, R.P of China.
[2]Heyuan Polytechnic, Heyuan 517000, Guangdong, China
[3]Southern Marine Science and Engineering Guangdong Laboratory, Guangzhou, 511458, China
# These authors contributed equally to the work.

*Correspondence author: Zhong Hu, Email: hzh@stu.edu.cn

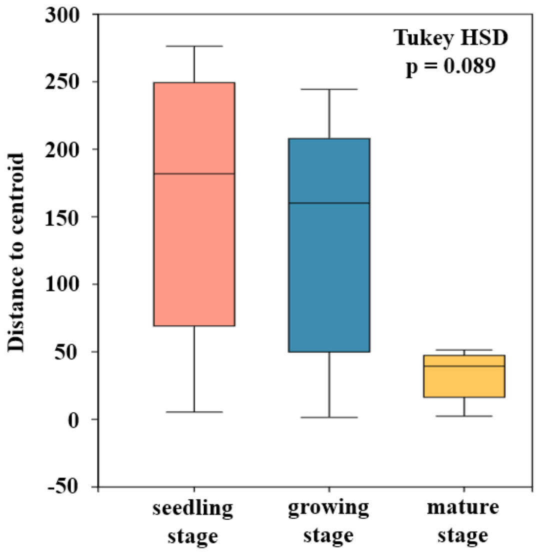

Supplemental Figure S1. The beta diversity of community across different growth stages. The difference of surface communities at maturity stage was smaller compared with the seedling stage (P = 0.089).

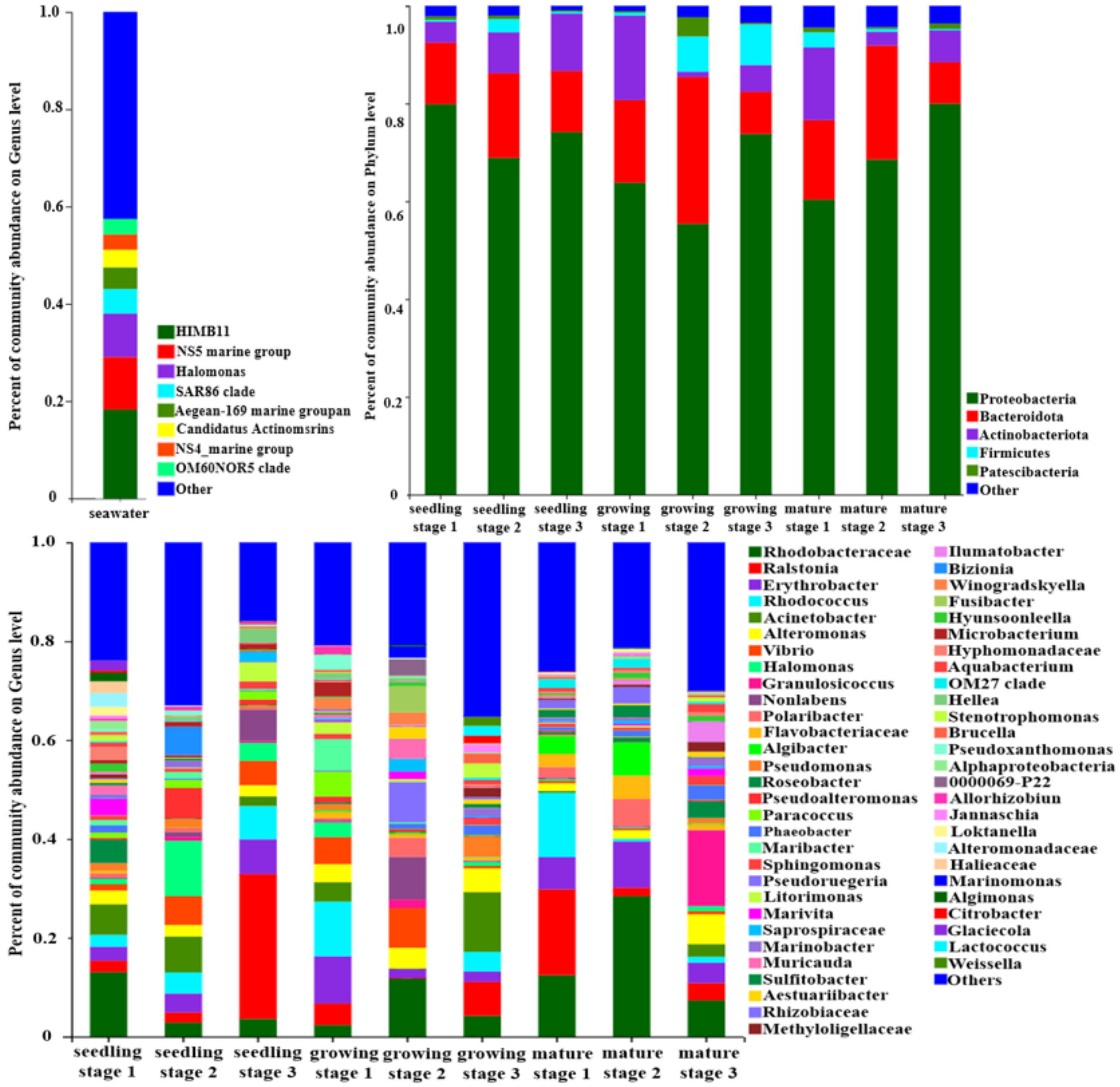

Supplemental Figure S2. Changes of relative abundance (%) of bacterial community on the surrounding seawater **(A)** and on the surface of *U.fasciata* at phylum level **(B)** and at the genus level **(C)** during the growth different stage. (Sea water samples in different stages are relatively similar, figure A shows the average value of all sea water samples)

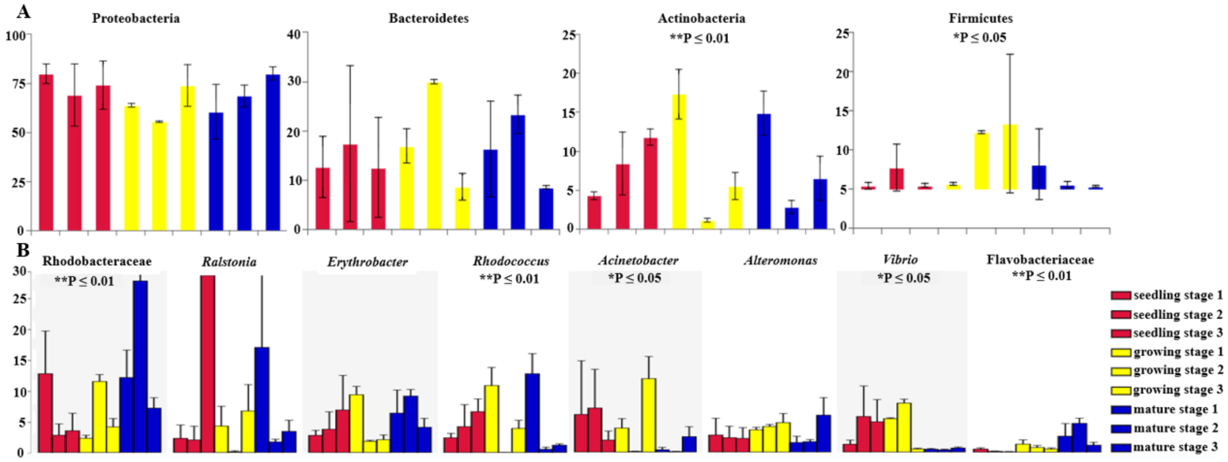

Supplemental Figure S3. Used the Kruskal-wallis test to calculate the relative abundance of different bacterial species at phylum and genus levels with different growth stages. (Data are presented as means standard errors (n=3). *, P<0.05, **, P<0.01, ***, P<0.001)

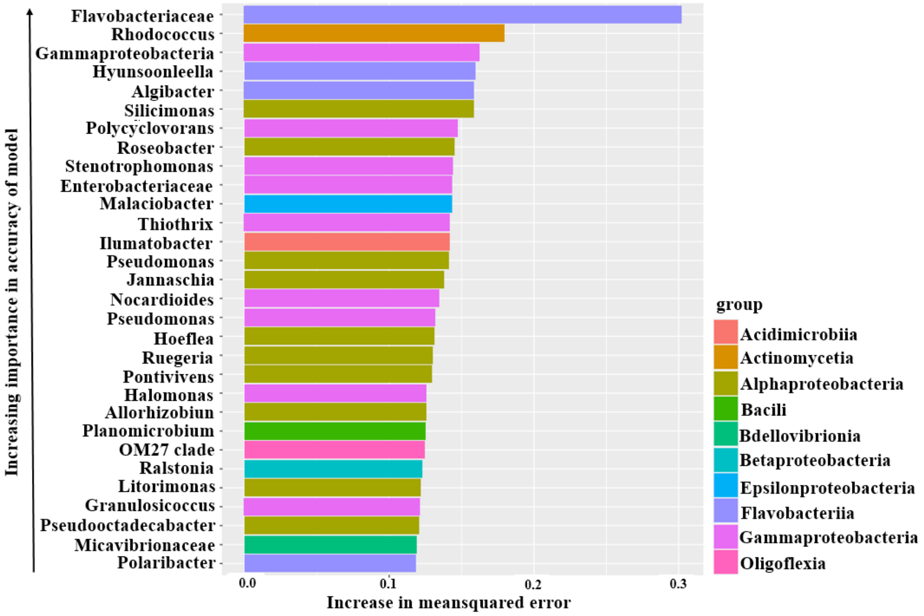

Supplemental Figure S4. Biomarkers of *U. fasciata* for horizontal classification of bacterial genera at different growth stage. Using random forest regression method, the relationship between the relative abundance of bacteria in different growth stage and the growth stage of *U. fasciata*

were analyzed. There are 30 biomarker bacteria were identified. The accuracy of the model was influenced by the order of importance of biomarker taxa.

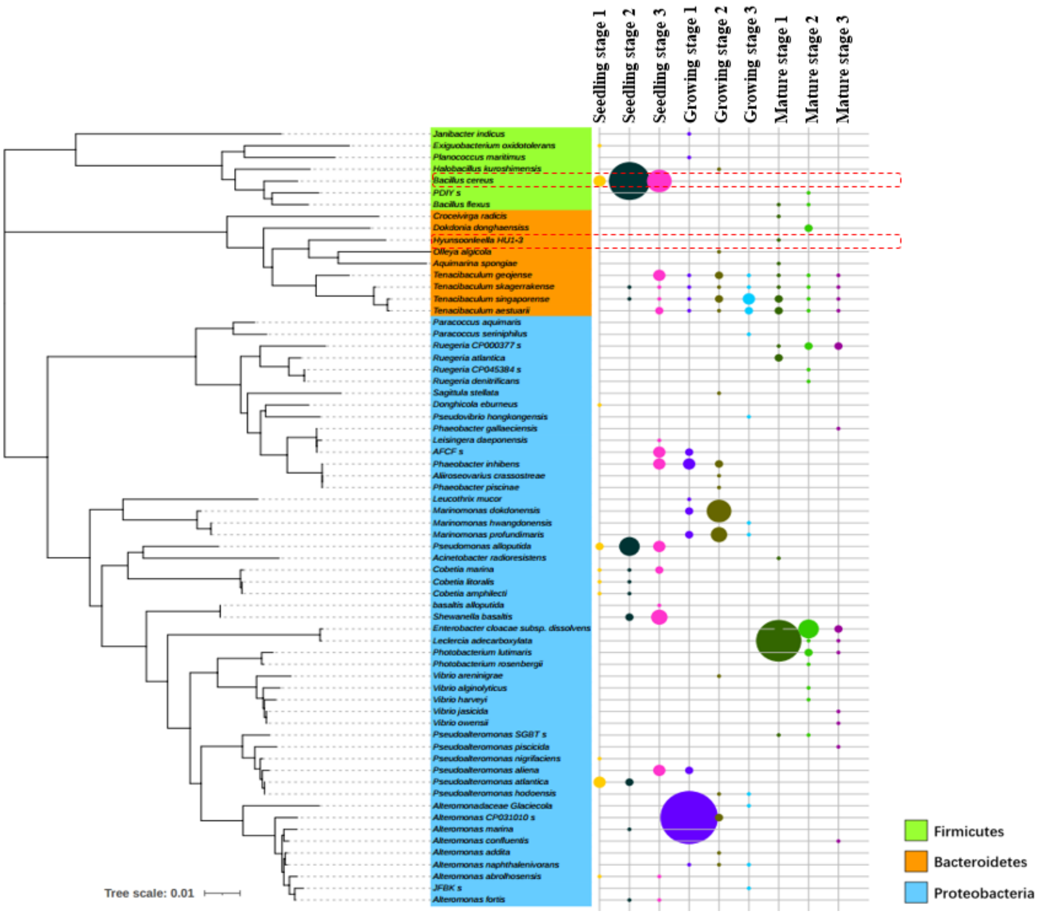

Supplemental Figure S5. Evolutionary relationship and number of strains screened from the surface of *U.fasciata* at different growth stages.（Neighbour-joining phylogenetic tree based on 16S rRNA gene sequences showing the positions of strain, scale bar 0.01 substitutions per nucleotide position. The size of the circle is proportional to the number of strains）

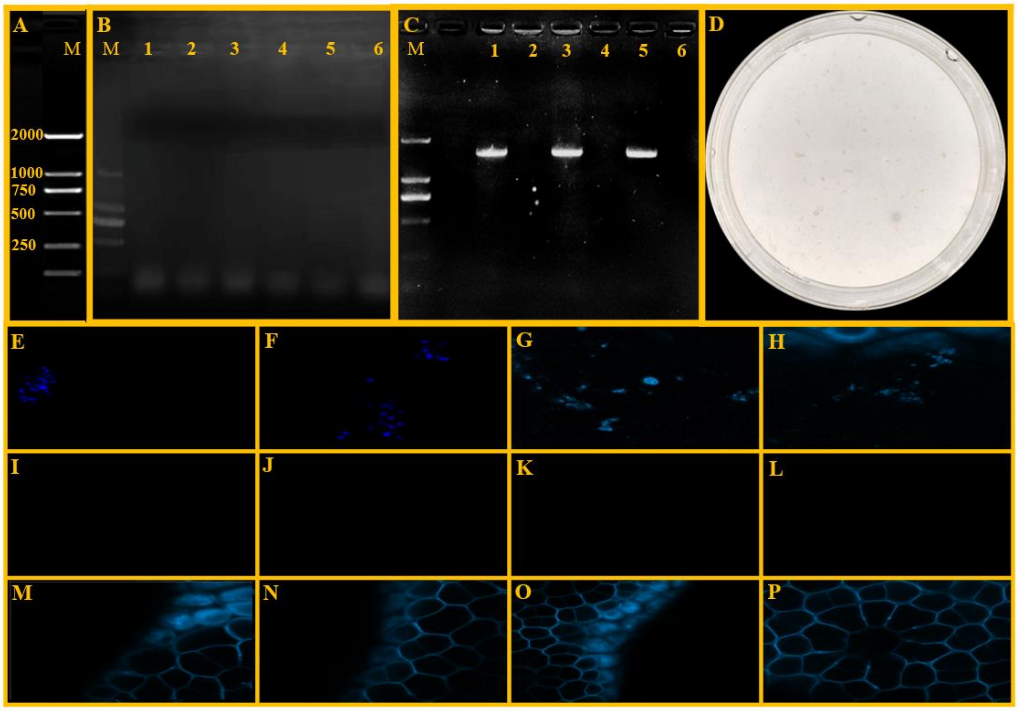

Supplemental Figure S6. Aseptic validation of *U. fasciata* (SBR). **(A)** stripe map of bacterial DNA Marker 2000, **(B)** the seawater collected during the last rinse as a template, and the universal primer of bacteria was used to amplifie the full length 16S rDNA by PCR（lanes 1-6 are parallel experiments, and no PCR products were obtained at 1500bp, which proved that no bacteria existed.）, **(C)** *U. fasciata* (SBR) was ground into liquid, then this liquid was used as template to complete the amplification of full-length 16S rDNA (lanes 1,3,5 are parallel experiments, PCR products were found at 1500bp which confirming the presence of bacteria inside the *U. f*asciata (SBR)), **(D)** the seawater collected at the time of the last rinse was coated with ocean 2216E plate without bacterial strain growth after one week, **(E-H)** *U. fasciata* was stained with 1% (V/V) DAPI and observed by confocal laser scanning microscope （CLSM）, and large numbers of bacterias were found, **(I-L)** *U. fasciata* (SBR) was stained with 1% (V/V) DAPI and examined by CLSM，there was no bright blue spots be observed which indicating the absence of bacteria, **(M-P)** *U. fasciata* (SBR) was stained with 1% (V/V) DAPI and observed by CLSM，and few bacteria can be found in the intercellular space *Ulva* cells.

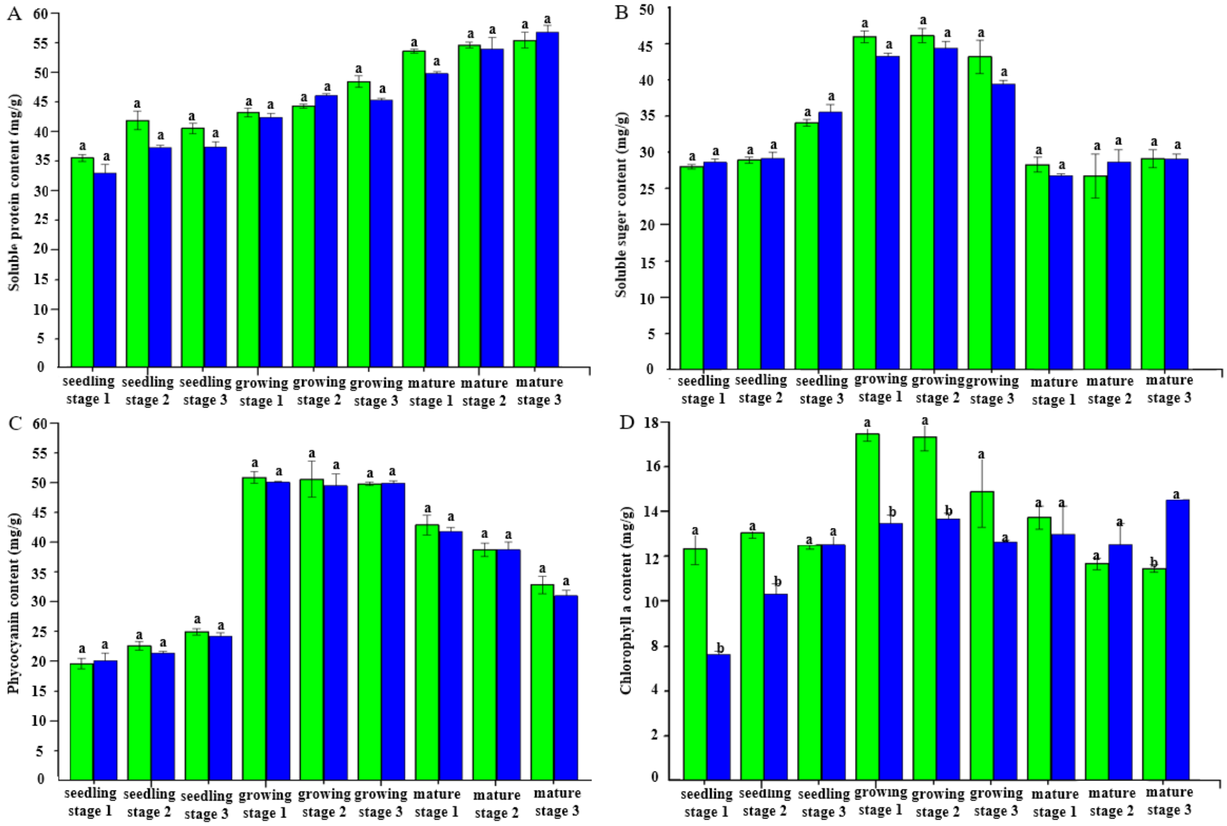

Supplemental Figure S7. Comparison of physiological indexes before and after surface sterilization of *U.fasciata* at different growth stages. **(A)** Soluble protein **(B)** Soluble suger **(C)** Soluble suger **(D)** Chlorophyll a (The green represents the samples of *U.fasciata* in environment and the blue represents the samples of *U.fasciata* after sterilization. The difference coefficients a and b are the differences between the samples of *U.fasciata* in the same batch)

Supplemental Table S1: Different physical environmental parameters of the sampling samples.

**Sample**	**Temp (℃)**	**DO (ppm)**	**pH**	**Salinity (‰)**
seedling stage1	22.57±0.03	89.63±0.34	8±0	32.67±0.33
seedling stage2	21.5±0.06	85.63±3.17	8.1±0	33±0
seedling stage3	20.73±0.15	88.53±0.24	8.1±0	33±0
growing stage1	18.2±0.12	89.27±0.41	8.1±0	34±0
growing stage2	18.47±0.03	87.37±0.48	8.13±0.03	32±0
growing stage3	18.63±0.15	88.3±1.06	8.07±0.03	32.67±0.33

mature stage1	19.47±0.09	93.8±0.96	8.07±0.03	32.67±0.33
mature stage2	20.07±0.03	83.13±0.29	8.07±0.03	32.67±0.33
mature stage3	22.13±0.09	82.57±0.32	7.9±0	34.67±0.33

Supplemental Table S2: Enzymes involved in plant hormone production/regulation in genome of strain *Hyunsoonleella* sp. HU1-3.

genetic code	genetic name	gene function
JKAMIBLE_0071	Tryptophan 2,3-dioxygenase	IAA production
JKAMIBLE_0117	Tryptophan--tRNA ligase	
JKAMIBLE_0228	Tryptophan synthase beta chain	
JKAMIBLE_0228	Tryptophan synthase alpha chain	
JKAMIBLE_0259	Tryptophan-rich sensory protein	
JKAMIBLE_0032	N-acetylmuramoyl-L-alanine amidase LytC	
JKAMIBLE_0046	2-oxoglutaramate amidase	
JKAMIBLE_0262	Mycothiol S-conjugate amidase	
JKAMIBLE_0269	N-acetylmuramoyl-L-alanine amidase AmiC	
JKAMIBLE_0272	Bifunctional glutathionylspermidine synthetase/amidase	
JKAMIBLE_0339	tRNA dimethylallyltransferase	Cytokinin production
JKAMIBLE_0102	putative tRNA-dihydrouridine synthase	
JKAMIBLE_0217	tRNA-2-methylthio-N(6)-dimethylallyladenosine synthase	
JKAMIBLE_0268	tRNA N6-adenosine threonylcarbamoyltransferase	
JKAMIBLE_0339	tRNA dimethylallyltransferase	
JKAMIBLE_0137	3',5'-cyclic adenosine monophosphate phosphodiesterase CpdA	
JKAMIBLE_0123	Cytokinin riboside 5'-monophosphate phosphoribohydrolase	
JKAMIBLE_0087	3',5'-cyclic adenosine monophosphate phosphodiesterase CpdA	
JKAMIBLE_0151	Bifunctional cytochrome P450/NADPH--P450 reductase 1	
JKAMIBLE_0093	NAD(P)-specific glutamate dehydrogenase	
JKAMIBLE_0258	NAD-specific glutamate dehydrogenase	
JKAMIBLE_0043	Iron-sulfur cluster carrier protein	Iron carrier
JKAMIBLE_0055	Fumarate reductase iron-sulfur subunit	
JKAMIBLE_0056	Iron-sulfur cluster insertion protein ErpA	
JKAMIBLE_0086	Menaquinone reductase, iron-sulfur cluster-binding subunit	
JKAMIBLE_0094	Iron-dependent repressor IdeR	
JKAMIBLE_0327	Menaquinone reductase, iron-sulfur cluster-binding subunit	
JKAMIBLE_0000	Pyridoxine/pyridoxamine 5'-phosphate oxidase	Phosphate protein
JKAMIBLE_0001	Isopentenyl-diphosphate Delta-isomerase	
JKAMIBLE_0002	Phosphoglycolate phosphatase	
JKAMIBLE_0008	Carbamoyl-phosphate synthase large chain	

JKAMIBLE_0009	Dodecaprenyl-phosphate galacturonate synthase
JKAMIBLE_0013	Mannosylfructose-phosphate synthase
JKAMIBLE_0013	Mannosylfructose-phosphate phosphatase
JKAMIBLE_0017	Aminoalkylphosphonate N-acetyltransferase
JKAMIBLE_0025	Phospho-N-acetylmuramoyl-pentapeptide-transferase
JKAMIBLE_0044	Alkaline phosphatase synthesis sensor protein PhoR
JKAMIBLE_0044	Alkaline phosphatase synthesis transcriptional regulatory protein PhoP
JKAMIBLE_0048	Pyridoxine 5'-phosphate synthase
JKAMIBLE_0051	Thiamine-phosphate synthase
JKAMIBLE_0052	Geranylgeranylglyceryl phosphate synthase
JKAMIBLE_0056	Thiamine-monophosphate kinase
JKAMIBLE_0066	Phosphoglycolate phosphatase
JKAMIBLE_0085	Sucrose 6(F)-phosphate phosphorylase
JKAMIBLE_0103	Alpha-maltose-1-phosphate synthase
JKAMIBLE_0117	1-acyl-sn-glycerol-3-phosphate acyltransferase
JKAMIBLE_0121	Histidinol-phosphatas
JKAMIBLE_0134	Glucosamine-6-phosphate deaminase 1
JKAMIBLE_0139	Alkaline phosphatase synthesis transcriptional regulatory protein PhoP
JKAMIBLE_0140	Alkaline phosphatase synthesis sensor protein PhoR
JKAMIBLE_0154	L-fuculose phosphate aldolase
JKAMIBLE_0256	Alkaline phosphatase PafA
JKAMIBLE_0324	Alkaline phosphatase synthesis transcriptional regulatory protein PhoP
